# The Role of ChatGPT in the Advancement of Diagnosis, Management, and Prognosis of Cardiovascular and Cerebrovascular Disease

**DOI:** 10.3390/healthcare11212906

**Published:** 2023-11-06

**Authors:** David-Dimitris Chlorogiannis, Anastasios Apostolos, Anargyros Chlorogiannis, Leonidas Palaiodimos, George Giannakoulas, Sumant Pargaonkar, Sofia Xesfingi, Damianos G. Kokkinidis

**Affiliations:** 1 Department of Radiology, Brigham and Women’s Hospital, Boston, MA 02115, USA; 2 First Department of Cardiology, School of Medicine, National Kapodistrian University of Athens, Hippokrateion General Hospital of Athens, 115 27 Athens, Greece; anastasisapostolos@gmail.com; 3 Department of Health Economics, Policy and Management, Karolinska Institutet, 171 77 Stockholm, Sweden; 4 Division of Hospital Medicine, Jacobi Medical Center, NYC H+H, Albert Einstein College of Medicine, New York, NY 10461, USA; leonidas.palaiodimos@nychhc.org (L.P.); sumant.pargaonkar@gmail.com (S.P.); 5 Department of Cardiology, AHEPA University Hospital, Aristotle University of Thessaloniki, 541 24 Thessaloniki, Greece; g.giannakoulas@gmail.com; 6 Department of Economics, University of Piraeus, 185 34 Piraeus, Greece; 7 Section of Cardiovascular Medicine, Yale University School of Medicine, New Haven, CT 06510, USA

**Keywords:** cardiovascular disease, cerebrovascular disease, chatGPT, language-based models, diagnosis, treatment, stroke, myocardial infarction

## Abstract

Cardiovascular and cerebrovascular disease incidence has risen mainly due to poor control of preventable risk factors and still constitutes a significant financial and health burden worldwide. ChatGPT is an artificial intelligence language-based model developed by OpenAI. Due to the model’s unique cognitive capabilities beyond data processing and the production of high-quality text, there has been a surge of research interest concerning its role in the scientific community and contemporary clinical practice. To fully exploit ChatGPT’s potential benefits and reduce its possible misuse, extreme caution must be taken to ensure its implications ethically and equitably. In this narrative review, we explore the language model’s possible applications and limitations while emphasizing its potential value for diagnosing, managing, and prognosis of cardiovascular and cerebrovascular disease.

## 1. Introduction

Cardiovascular and cerebrovascular disease are still the leading cause of premature morbidity, mortality, and disease disability on a global scale [1]. Despite significant advancements in the standard of care, particularly in higher-income countries, the incidence of these diseases continues to rise [2]. This finding has been partially attributed to poorly controlled preventable risk factors such as hypertension, hyperlipidemia, smoking, diabetes, and obesity, the latter now being widely considered a pervasive epidemic of the 21st century, despite the significant development of newer pharmaceutic agents for primary and secondary prevention, improved invasive procedures, and better diagnostic tools [3].

In this era of rapid technological progress, artificial intelligence (AI) has emerged as a central pillar of the fourth industrial revolution. Artificial intelligence encompasses a field of computer science in which machines are trained to perform tasks that typically require human intelligence. AI has already gained ground in radiology, where it helps with more accurate image processing and interpretation [4]. The concept of digital symptom checking is not new and began in the early 2000s with WebMD, which provided a handful of risk calculators available to the public [5,6]. The days of the binary calculators have long past and ever since there has been a rapid development of more than ten artificial intelligence models, each demonstrating remarkable capabilities [7]. Since primary prevention and risk factor modification play a pivotal role in the management of cardiovascular and cerebrovascular disease, there has been a surge of interest in artificial intelligence’s role, especially for language-based models, in the advancement of cardiovascular and cerebrovascular disease management and financial burden reduction. One such language-based model, ChatGPT, is at the forefront of this artificial intelligence revolution [8].

ChatGPT is an artificial intelligence language-based model developed by OpenAI and was based on the Generative Pre-trained Transformer (GPT) architecture, specifically the GPT-4 version [8]. GPT-4 version is a subtype of machine learning algorithms and more specifically a neural network, trained on a vast amount of internet text, whose primary functions are to understand, generate human-like text, and perform a variety of natural language processing tasks, depending on the input provided. The input can be a word or text and then the model “breaks down” the input to smaller parts and analyzes it. Then, through statistical computations, it generates an output. These include text generation, language translation, text summarization, sentiment analysis, named entity recognition, and question answering. The model first launched on 30 November 2022, and quickly divided the scientific community with its capabilities [9,10], ranging from simple student essay generation to research articles, with submissions even including ChatGPT as a co-author [11]. Less than a year after its release, more than 1200 results appeared after using ChatGPT as a keyword in searches, showing its significant impact on current medical research. The extent of its abilities prompted essential questions regarding its potential application in clinical practice, specifically its power to revolutionize critical medical fields such as cardiovascular and cerebrovascular disease management. The primary objective of this narrative review article is to explore the potential role of ChatGPT in those fields.

## 2. Search Methods

A comprehensive literature review was meticulously conducted for published articles focused on using ChatGPT in cardiovascular and cerebrovascular diseases. MEDLINE database via PubMed was used to collect relevant articles. A search set combined the keyword “ChatGPT” to gather all the relevant papers on this topic, and no language restriction was used. Using this approach, all articles related to ChatGPT’s application in cerebrovascular and cardiovascular disease were collected. Overall, this study analyzed the potential application of ChatGPT for the prompt diagnosis, prognosis, and management of patients with cardiovascular and cerebrovascular disease by considering various published articles.

## 3. ChatGPT for Diagnosis of Cardiovascular and Cerebrovascular Disease

Cardiovascular and cerebrovascular disease manifest from multifaceted complex pathophysiological etiologies, having been linked with multiple risk factors [12]. Given their extremely high impact on morbidity and mortality associated with their prevalence, particular emphasis is required on risk factor modification as a primary means of reducing their incidence. In this context, we initiated a conversation with ChatGPT to explore its potential as a diagnostic tool. The response of the model elucidated several critical instances through which ChatGPT can enhance the diagnosis of these diseases:

ChatGPT can be used to advance the diagnosis of cardiovascular disease and stroke by analyzing patient data and providing personalized recommendations to healthcare providers, leveraging its natural language processing and machine learning capabilities. For this reason, we asked the ChatGPT “how it can be used to improve the diagnosis of cardiovascular disease and cerebrovascular disease”:

*Symptom analysis: ChatGPT can assist in analyzing patient-reported symptoms related to cardiovascular issues or stroke. Patients often describe their symptoms in plain language, and ChatGPT can understand and interpret these descriptions. For example, if a patient reports symptoms such as chest pain, shortness of breath, or sudden weakness on one side of the body, ChatGPT can analyze this information and provide insights to healthcare providers. This can help identify potential cardiovascular or stroke-related issues early in the diagnostic process.*

*Differential diagnosis: ChatGPT can aid healthcare providers in generating a comprehensive list of potential diagnoses based on a patient’s reported symptoms and medical history. By cross-referencing the patient’s information with a vast database of medical knowledge and guidelines, ChatGPT can help rule out non-cardiovascular or non-stroke-related conditions and provide a more accurate differential diagnosis. This supports healthcare professionals in making informed decisions about further diagnostic tests and evaluations.*

*Risk assessment: In addition to symptom analysis, ChatGPT can evaluate other risk factors associated with cardiovascular disease and stroke. It can consider a patient’s age, gender, family history, lifestyle factors (such as smoking and diet), and relevant medical history (such as diabetes or hypertension). ChatGPT can provide insights into a patient’s overall risk of developing cardiovascular disease or experiencing a stroke by assessing these risk factors. This information helps in risk stratification and informs the diagnostic process*.

*Real-time data collection: ChatGPT can collect and analyze patient data during interactions. For example, suppose a patient is experiencing acute symptoms, such as sudden chest pain or neurological deficits. In that case, ChatGPT can prompt the patient to provide additional information about the symptoms, their duration, and any associated factors. This real-time data collection can aid healthcare providers in making prompt and accurate diagnostic decisions, particularly in emergency situations.*

*Referral recommendations: Based on the analysis of symptoms and risk factors, ChatGPT can suggest appropriate referrals to other healthcare providers or specialists. For instance, if a patient’s symptoms indicate a potential heart condition, ChatGPT can recommend a cardiology consultation or additional cardiac tests. These referral recommendations help ensure that patients receive timely and appropriate care from the right specialists.*

*Education and Patient Empowerment: ChatGPT can educate patients about the importance of early diagnosis and seeking medical attention for symptoms related to cardiovascular disease or stroke. It can explain the significance of diagnostic tests, such as electrocardiograms (ECGs), echocardiograms, or brain imaging, and address any concerns or misconceptions patients may have. By empowering patients with knowledge, ChatGPT can encourage them to proactively engage in the diagnostic process.*

*Support for Telemedicine: In the era of telemedicine, ChatGPT can serve as a virtual assistant during remote consultations with healthcare providers. It can help patients describe their symptoms accurately and provide healthcare professionals with essential information for remote diagnosis. This enhances the accessibility of healthcare services, especially for patients in remote or underserved areas.*

*In summary, ChatGPT’s role in the advancement of the diagnosis of cardiovascular disease and stroke is multifaceted. It contributes to symptom analysis, supports differential diagnosis, assesses risk factors, collects real-time data, facilitates referral recommendations, educates patients, and enhances telemedicine interactions. By assisting healthcare providers in the diagnostic process, ChatGPT plays a valuable role in the early detection and management of these critical medical conditions, ultimately leading to better patient outcomes.*

Regarding diagnosis for cardiovascular and cerebrovascular disease, its main strengths are the system’s capacity to offer instantaneous responses regarding symptom analysis, risk assessment, real-time data collection, differential diagnosis aid, referral recommendations, education and patient empowerment, and support for telemedicine. Indeed, language-based models with a simple chatbox-like interface can analyze the symptoms experienced by the patient or other inserted data as input and, after examining them, return a result based on the patient’s disease risk. Against this background, Rizwan and Sadiq explored the diagnostic accuracy and accessibility of ChatGPT in cardiovascular diseases compared to experts in clinical cardiology and imaging. Eight of the ten clinical scenarios processed by ChatGPT were perfectly diagnosed; however, the other two answers given by ChatGPT were partially incorrect since those diseases were associated with the actual diagnosis. Moreover, the therapeutic protocols that ChatGPT suggested in the same study were in accordance with the literature and current medical knowledge [13]. In addition, Kusunose et al. investigated the ability of ChatGPT to answer clinical questions stated in the Japanese Society of Hypertension Guidelines. ChatGPT showed a high accuracy rate, but it varied by available evidence [14]. This ability could be enhanced by incorporating the 10-year risk prediction algorithms for cardiovascular disease estimates, like the SCORE2 for the European population and SCORE2-OP for the older population in four geographical risk regions [15,16]. Thus, it can aid in the early detection of high-risk populations and, by analyzing individualized data, refer them promptly to primary prevention care. This feature can be crucial and lifesaving, especially when prompt decisions are imperative, such as determining the need for reperfusion strategies. In addition, artificial intelligence models have been specifically trained to process large amounts of data in much less time than would otherwise require a person. Thus, it may facilitate not only data extraction from large healthcare datasets for research purposes without the risk of human error but also in generating succinct patient medical records or patient history summaries, which are essential for more effective patient management.

However, this is not the only utilization of ChatGTP in medical research. Recently, Teperikidis et al. performed an umbrella review and searched for systematic reviews and meta-analyses to answer the research question of whether there is a causal relationship between protein pump inhibitor administration and major adverse cardiovascular events [17,18]. The originality of this project was not the main focus; rather, the utilization of ChatGTP in the automated process of this research project was the objective. Two independent reviewers performed the systematic search, while a second pair of investigators performed the same task by exclusively using ChatGPT. Interestingly, the studies utilizing ChatGPT were consistent with the conventional manual approach, adding another artificial intelligence capability in medical research [17,18]. Furthermore, there is great potential in other areas of medicine, like patient education. The interaction of patients with the language-based model could provide the initial motivation to get referred to the appropriate healthcare specialist or emergency department. Moreover, numerous patients seek medical advice in search engines, read inappropriate articles, and adopt symptoms and signs, which drives them to wrong clinical self-diagnosis and stresses them. In this context, ChatGPT could also play an advisory role to these patients, as its recommendations will be more reliable, taking into consideration that such advice is more personalized and evidence based [19]. ChatGPT differs from other search engines, meaning that it does not always produce the same answer because each of the model’s responses can vary depending on how the user formed the question [20].

Furthermore, the ability to simplify difficult-to-understand pathophysiological concepts or procedures while also being readily accessible makes it a solid option for patient education armamentarium. In this context, Khan et al. explored the capacity of the model to hold conversations about diabetes in a casual manner. They found that its responses were scientifically sound and easy to interpret, signifying the potential to extrapolate this ability with other diseases [21]. Similarly, Haver et al. tested the appropriateness of the responses of the model for Breast Cancer prevention recommendations and found that the responses of the model were appropriate 22 out of 25 times [22]. In the cardiovascular setting, the model was tested in 25 questions concerning core preventive concepts, including risk factor counseling, test results, and medication information, based on guideline-based prevention topics. The authors found that the responses were appropriate in 21 cases, highlighting the need for improvement due to the risk for misinformation [23]. In addition, these responses can even be tailored to patients’ specific educational levels and replace the general written materials that utilize a general text for all. Although tested in a different setting than cardiovascular diseases, ChatGPT’s role in generating patient education materials was examined by Hung et al. in the vicinity of plastic surgery and compared to ones created by experts [24]. The authors found that ChatGPT’s education materials were generated in 30 min, while the content generated by experts took approximately one month. However, this came at the cost of lower accuracy (50%) and readability of its content measured using the Flesch–Kincaid score (10.5 grade versus 7.5 grade, respectively), stressing that there is still room to grow. Chiwon Ahn et al. explored whether ChatGPT can provide reliable information regarding cardiopulmonary resuscitation, which is a topic of general interest and a life-saving procedure for the general population. The authors noted that although ChatGPT cannot provide visual feedback to help readers comprehend the necessary steps, the answers it offered were descriptive and scientifically correct, adding to its potential for general medical educational purposes for a significant public health issue [4].

## 4. ChatGPT as a Tool for Secondary Prevention and Management

Moreover, the promising future of ChatGPT in primary prevention strategies has also fueled the interest in its potential value as a cardiovascular and cerebrovascular disease management and prognostic tool (secondary prevention). We began a second conversation with ChatGPT, asking if it “can be used to advance the management and prognosis of cardiovascular disease and stroke”. Its response was as follows:

*Chat GPT can be used to advance the management and prognosis of cardiovascular disease and stroke in several ways, leveraging its natural language processing and machine learning capabilities. Here are some specific ways in which Chat GPT can help healthcare providers to manage and predict outcomes for patients with cardiovascular disease and stroke:*

*Risk stratification: ChatGPT can analyze patient data, including medical history, lifestyle factors, and diagnostic test results, to provide risk stratification for individuals with CVD or stroke. By assessing various risk factors such as age, gender, family history, smoking status, and cholesterol levels, ChatGPT can help healthcare providers identify patients who are at higher risk of adverse outcomes. This risk stratification enables healthcare teams to allocate resources more effectively and provide more intensive management for high-risk patients.*

*Treatment optimization: ChatGPT can assist healthcare providers in optimizing treatment plans for patients with CVD or stroke. It achieves this by analyzing patient data and generating personalized treatment recommendations. These recommendations take into account the patient’s medical history, comorbidities, and medication preferences. By tailoring treatment plans to individual patients, ChatGPT helps healthcare providers identify the most effective interventions, reducing the risk of adverse events and optimizing outcomes.*

*Medication management: Ensuring medication adherence is crucial in managing CVD and stroke. ChatGPT can play a vital role in helping patients manage their medication regimens effectively. It can provide patients with reminders for medication adherence, explain the importance of each medication, and address any concerns or questions patients may have about their prescribed medications. By improving medication adherence, ChatGPT contributes to better disease management and outcomes.*

*Symptom management: Patients with CVD or stroke often experience symptoms that require ongoing management. ChatGPT can assist healthcare providers in managing these symptoms by analyzing patient-reported data and providing personalized recommendations. For instance, if a patient reports symptoms like chest pain or shortness of breath, ChatGPT can offer guidance on symptom management strategies, helping patients improve their quality of life and reduce the risk of complications.*

*Predictive modeling: Chat GPT can help healthcare providers predict outcomes for cardiovascular or stroke patients. By analyzing patient data and identifying patterns, Chat GPT can help healthcare providers predict the likelihood of adverse effects and take proactive steps to prevent them.*

*Rehabilitation Support: Patients often require rehabilitation and lifestyle adjustments after experiencing a cardiovascular event like a heart attack or stroke. ChatGPT can assist in providing guidance on post-event rehabilitation programs, exercise routines, dietary changes, and lifestyle modifications. It can offer personalized recommendations to help patients recover and reduce the risk of future cardiovascular events.*

*Patient Education: Educating patients about their condition, treatment options, and lifestyle modifications is essential for effective disease management. ChatGPT can serve as a valuable educational resource, providing patients with clear and accessible information. It can explain complex medical concepts, clarify treatment plans, and address patient queries, empowering patients to actively participate in their care.*

*In summary, ChatGPT’s role in the management and prognosis of cardiovascular disease and stroke lies in its ability to provide personalized, data-driven insights and recommendations. By leveraging patient data and medical knowledge, ChatGPT enhances risk assessment, treatment optimization, medication management, symptom control, predictive modeling, rehabilitation support, and patient education. This comprehensive approach contributes to better patient outcomes, improved quality of care, and more effective management of these critical medical conditions.*

For the management and prognosis of cardiovascular and cerebrovascular disease, the system’s value is, again, in providing instant and personalized questions after analyzing individualized data. The accelerated response time of the “heart or brain team” for reperfusion strategies can be lifesaving. At the same time, the model may also declare if the patient is eligible for inclusion in a trial and manage accordingly [25]. Moreover, since language-based models have already been considered a powerful tool in medical education [26], they may be replicated in patient education as well. Medication nonadherence has been linked with poor patient and caregiver communication [23,24,27], and language-based models, with their natural text, can provide knowledge regarding the benefits and reasoning behind treatment plans while also answering patients’ questions at any time, even providing symptomatic treatment recommendations during the rehabilitation period after the event. Lastly, the enhanced data processing may further aid in identifying patients’ unique clinical characteristics and thus suggest individualized treatment plans, facilitating the transition from evidence-based medicine to a more personalized one while also recognizing the increased likelihood for drug-to-drug interactions (DDIs) when proposing such a plan.

## 5. Discussion and Future Directions

An overall representation of the possibilities of ChatGPT in the clinical setting is depicted in Table 1. Even though the future of language-based models in medical care and research looks promising, it is of utmost importance to keep in mind several risks and concerns. Firstly, it is worth noting that the current ChatGPT version has been trained with data available up to 2021. Thus, it may need to be updated to keep up with the ever-changing medical landscape, deeming some of its recommendations not up to date. Furthermore, the inherent over-dependence on the training data has raised concerns about bias introduction, its external validity [25], and the potential risk of incomplete or inaccurate results. Furthermore, people who were excluded from the training dataset that the AI algorithms have trained with may lead to diagnosis failures and thus discriminate against them. An example was published by Kamulegeya et al. who showed that in an AI model with skin lesion classification was different between patients with different skin color. The training data was comprised of images from roughly 90% white people and 5–10% from other ethnicities and as a result the diagnostic accuracy in this population was half [28]. In this context, one of the main limitations of the language-based system is the hallucination bias, where the system reports a scientifically sound answer that cannot be realistically implemented [29]. This is a particular problem since it may produce fabricated information only to provide an answer to the user. For example, if the user asks the ChatGPT “who the sole survivor of the Titanic was” the model will reply “*The sole survivor of the Titanic disaster was Charles Joughin*”, when in reality there were 706 total survivors. Moreover, the model has a non-deterministic structure, meaning that the same input may result in different answers, hindering its classification capabilities. Thus, before implementation in the current evidence-based care system, several universal standards must be secured, like bias minimization, reproducibility, and accuracy of the results. In this domain, cross-validation of the information provided by ChatGPT must always be cross-checked with up-to-date human-annotated references and guidelines, since they are often updated and enhance the levels of their recommendations [30,31]. Additionally, institutions and expert healthcare providers must solidify the model’s unbiased responses while proving its benefit in the contemporary evidence-based medicine system with carefully planned research studies and legislating the context that the generated text follows a standardized reporting rule and does not misinform its users.

Lastly, its diagnostic deductive capabilities need to be more consistent and reproducible. In a study by Kung et al., the performance of ChatGPT was evaluated on the United States Medical Licensing Examination, and the language model performed at or near the passing threshold [32]. In another study, Skalidis et al. showed a similar performance of ChatGPT in the European Exam in Core Cardiology, as it answered correctly at about 60%, considered a passing score. Nevertheless, it could have been more efficient in the American Heart Association Basic Life Support and Advanced Cardiovascular Life Support exams. In this study, ChatGPT failed to reach the passing threshold for any of the exams performed; however, its answers were promising due to being more detailed than the rationale provided by the Advanced Cardiovascular Support Committee [33]. Additionally, in a cross-sectional study by Sinha et al. [34], the capabilities of ChatGPT in solving higher-order reasoning were evaluated. One hundred random questions, including cardiovascular disease, were used from different pathology and internal medicine areas. The system’s accuracy reportedly reached 75%, also underlining the room to grow in clinical decision-making. The model’s inaccurate responses or even worse false positive diagnoses may lead to catastrophic vents for the patients. The overreliance to the AI should be mitigated by rigorous validation and physician oversight [35]. Of note, a physician must not only rely on the initial response of the model, since medicine and diseases have a dynamic course, and the state of the patient may rapidly deteriorate. Thus, real-time patient surveillance is indispensable for patient care. Finally, Wang et al. tackled another important aspect of the integration of AI in healthcare concerning the ethical and legal considerations [36]. The authors noted a clear legal gap for responsibility allocation for when patient harm may occur as well as data privacy breach. In order to overcome these potential issues, before the implementation of AI in the contemporary clinical practice, strict adherence to ethical standards must be prioritized.

Although artificial intelligence is in its early stages, there is undoubtedly unexploited potential not only for clinical practice but also for research [37]. Indeed, the role of language-based models in academics has been debated. Its ability to facilitate the writing efficacy, reducing the time required for manuscript preparation, and assisting with data collection, interpretation, and calculations, has also been tested in using tools for systematic reviews. Mahuli et al. [38]. tested the efficacy of ChatGPT in using risk-of-bias tools and for data extraction. The authors shared complete text and asked the language-based model for information that needed to be extracted. The authors found remarkable reproducible efficiency in both areas, underlining its potential to streamline these processes. Nevertheless, ChatGPT could improve the language and terminology used in scientific manuscripts and limit syntactical or grammatical errors without affecting the text’s original meaning.

Despite its several limitations, ChatGPT is not far from becoming indispensable for the modern generation of physicians. Although the users cannot upload an image in the application yet, it is believed that it will be one of the upcoming futures of a newer version of ChatGPT as with the version 4. By expanding on this ability, users could upload electrocardiograms, and the system could aid in their interpretation [39]. Artificial intelligence, with promising results, is already used for ECG interpretation. At the same time, some studies report that its diagnostic accuracy is better than cardiologists-experts in ECG interpretation [40]. In contrast, having been trained using data retrieved from large datasets, prediction modeling has been created to predict the risk of developing various cardiac diseases [40]. Adding to the imaging analysis potential, ChatGPT could even play a role in chest X-ray interpretation. At the same time, it is considered a fundamental imaging exam, with many stressing the need for every physician to acquire the skill of interpreting them. This is particularly useful in emergency departments, where X-ray misinterpretation may further drive clinical decision-making from the actual diagnosis. As such, numerous abnormal findings are still missed or misdiagnosed with high overall inter- and intra-variability between healthcare providers [34]. Thus, ChatGPT and AI-driven applications could improve clinical practice in the acute care setting, especially in primary healthcare centers, where general practitioners should take the first actions and guide the treatment plan, double-crossing the information provided by the language-based model.

## 6. Conclusions

In conclusion, the practice of medicine in the future will likely be influenced by the large language models like ChatGPT. The expanding potential uses for the diagnosis, management, and patient prognosis with high-incidence diseases like cardiovascular and cerebrovascular disease have definite potential through data extraction, high-risk population identification, and clinical decision-making. Nonetheless, as with every new technology it has limitations, and before implementation in evidence-based care, responsible and thorough evaluation with universally accepted standards must be conducted. AI procedures should be performed under physicians’ guidance to eliminate the possibility of language-model mistakes.

## Figures and Tables

**Table 1:** Potential ChatGPT Applications in Cardiovascular Disease Diagnosis and Treatment.

	ChatGPT Potential Capabilities
**Diagnostic Tool Potential**	Patient reported symptom analysis, Comprehensive differential diagnosis, Risk assessment, Real-time data collection, Referral Recommendations, Education and Patient Empowerment, Support for Telemedicine
**Treatment Tool Potential**	Risk stratification, Treatment optimization, Medication management, Symptom management, Predictive modeling, Rehabilitation Support, Patient Education

## Data Availability

Not applicable.
